# Addressing the Location Problem of a Perishables Redistribution Center in the Middle of Europe

**DOI:** 10.3390/foods10051091

**Published:** 2021-05-14

**Authors:** Juan Carlos Pérez-Mesa, M. Mar Serrano-Arcos, José Felipe Jiménez-Guerrero, Raquel Sánchez-Fernández

**Affiliations:** Department of Economics and Business, Mediterranean Research Center on Economics and Sustainable Development (CIMEDES), Agrifood Campus of International Excellence, CeiA3, University of Almería, 04120 Almería, Spain; marserrano@ual.es (M.M.S.-A.); jfjimene@ual.es (J.F.J.-G.); raquel.sanchez@ual.es (R.S.-F.)

**Keywords:** fruits and vegetables, waste, gravity center, gravity p-median, p-center, intermodal sustainable transport, uncertain location problem, multicriteria analysis

## Abstract

This work aims to contribute to the debate on practical utilization of different location models for consolidation, redistribution, and repackaging centers in a supply chain to optimize shipments, thereby reducing food loss and waste, within the framework of quality of customer service improvement. The scenario in question is the creation of a redistribution center for highly perishable products (fruits and vegetables) from southeast Spain—the leading European supplier—for customers throughout Europe. It is estimated that 10% of exports (more than 530,000 metric tons) from this area are returned by customers due to minor defects. These products cannot be reused and are therefore wasted. Regarding the methodology, comparisons were made between the p-median, gravity p-median, and p-center models. Scenarios of change in demand and randomness in distances were also tested. In addition, the modelling used included the cost and time within a multicriteria optimization framework to assess the possibility of a transport mode change. It was observed, for example, that the gravity p-median model proved useful for perishable products and the logistics strategy chosen. Furthermore, the p-median model displayed strong robustness against long-term changes in demand and random distances. In general, it was demonstrated that this strategy would successfully reduce the response time and distance of shipment from the distribution center to the customers and thereby improve sustainability of the service, reducing the waste related to direct shipments. Furthermore, this research also demonstrated the difficulty of using intermodality in this context, mainly due to transit time, which would undoubtedly increase the waste generated.

## 1. Introduction

The horticultural sector in southeast Spain is characterized by its vocation for exporting. For example, 40% of the vegetables consumed in Europe come from Spain. The importance of this sector is also reflected by its sales figures abroad: 5400 million euros [1], 97% of which come from the European Union (EU). At present, 72% of all the production is intended for exportation, mainly to Germany, France, and the United Kingdom. With respect to the distribution channel, it is important to highlight that the major European distribution chains (e.g., Aldi, Edeka, Tesco, Carrefour, and Lidl, among others) absorb 70% of the purchases from Spanish producers [2]. Despite these data, the commercialization system at the place of origin, which has made progress in switching from passive exportation to more active practices, has not taken any definitive action towards creating logistics platforms at places of destination in order to improve customer service [3] and the conditions (quality) of the products when they reach the final consumer. This article addresses the said issue while also attempting to adapt the classic location method to the specific case of perishables products, such as vegetables, which have been scarcely addressed in the literature (see, e.g., [4]). One of the characteristics of these products is that they require a more rigorous coordination of the supply chain to avoid production of waste. Therefore, logistics play a fundamental role in ensuring that products arrive in perfect condition to end customers by increasing their shelf life and the probability of being consumed and not wasted. In this sense, food loss and waste (FLW) occur at every stage of the food supply chain. Furthermore, there are two main costs associated with this loss: economic and environmental. The economic cost includes not only the loss of value of the products themselves, but also the costs linked to the production, transportation, and storage of the wasted products in addition to the handling costs. The environmental cost includes waste of the resources throughout the products’ life cycle, namely, land, water, energy, and other inputs, and the consequential increase in greenhouse gas emissions. The production stage represents 24–30% of the global FLW, while the post-harvest stage accounts for 20%, consumption—for 30–35%, being responsible for an estimated 8% of the Global Greenhouse Gas (GHG) emissions [5]. Specifically, 44% of the fruits and vegetables produced are FLW [6]. The localization strategies outlined in this article can help to solve this problem, especially when dealing with highly perishable products.

The distribution and location family of problems covers formulas that range in complexity from simple single commodity linear deterministic models to multicommodity nonlinear stochastic versions [7]. The techniques applied can be used for all types of facility location models, including single facility location, multiple facility location, quadratic assignment problems, location–allocation, covering problems, p-median problems, p-center problems, hierarchical facility location problem, hub location problems, competitive facility location, warehouse location problems, dynamic facility location problems, location inventory, location routing, location reliability and, recently, location in the supply chain, using a mix of different approaches [8].

**Figure 1:**
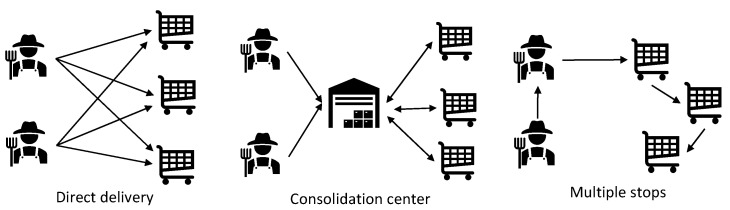
The principal merchandise distribution strategies between suppliers and customers. Source: own elaboration.

In this context, the general problem of locating distribution centers across a wide geographic area with several demand points remains an important theoretical and practical issue [9,10]. In short, merchandise can be distributed using different strategies [11]: (1) shipping products directly from suppliers to customers; (2) passing through a consolidation/deconsolidation or cross-dock platform; or even (3) following a multi-stop strategy. The present analysis proposes the creation of a logistics distribution and shipment consolidation center by applying reverse logistics (Figure 1) specifically to fruits and vegetables shipped from southeast Spain to the rest of Europe (the final destination of 99% of exports from this area). We considered the total amount supplied by the main vegetable-exporting provinces (including some fruits, such as melons and watermelons common to these areas) from southeast Spain. In order of importance: Almeria, Granada, and Murcia. In more general terms, it attempts to optimize the European horticultural supply chain through a more proactive logistics strategy by exporters.

In our case, the problem was addressed using the p-median, gravity p-median, and p-center methodologies due to their adaptation to our empirical study problem. The issue of allocating P facilities to Q demand points (geographically distributed) remains an important theoretical and practical problem. The p-median model minimizes the total distance traveled by consumers to facilities. This methodology has been widely tested in the literature [12,13]. The gravity p-median model is an important improvement on the widely used p-median model. However, there is still debate on its validity in empirical applications [14], which calls into question its potential applicability [9]. Nevertheless, in our specific case, certain peculiarities were identified that may prove useful, such as the distance decay parameter as a selection factor for choosing a location. The said characteristics may favor locations with an agglomeration of nearby destinations, which is convenient for perishable products. In addition, the p-center model is applied when it is necessary to ensure equity between the users served within a large geographic area [15]. This methodology has been applied to a wide variety of problems [16,17]. In our particular case, this aspect could prove to be of interest as it grants “flexibility” to the location chosen in case of possible sudden changes in customer demand, which is quite common in the horticultural sector.

With the aim of testing the robustness of the chosen unloading points, we modified the models utilized and assumed that the distances used in all the models were random variables. In the literature, various models exist which attempt to solve the facility location p-problem under uncertainty [18], basically by introducing risk in the volume transported [19,20] or even the future number of facilities [21]. Only Nikoofal and Sadjadi have introduced uncertainty in edge lengths that may appear in transportation costs or travel times along the edges in any network location p-problem [22].

In the models applied, we tried to locate the consolidation center in the facilities of an already existing customer in the supply chain. In this regard, it must be noted that the fundamental drawbacks are the construction and maintenance costs of these intermediate facilities, which may not compensate for the advantages of this strategy.

Among the reasons for creating this type of redistribution centers in Europe, specifically for perishable products (fruits and vegetables), are the following:

- Certain merchandise can be returned to the supplier for a variety of reasons. The said products could then be repackaged and served again to customers. In other words, the consolidation center would receive the defective produce (e.g., packaging in poor condition) so it may be reutilized. Alternatively, the products would be disposed of due to the impossibility of returning them to the place of origin. It is estimated that 10% of exports are returned by customers due to minor defects [23]. The said reason can be framed within reverse logistics, an area which has attracted considerable attention over the past decade (see, e.g., [24]). In economic terms, for the southeast of Spain, this represents more than 550 million euros of losses for exporters.
- In the case of the previous strategy, the response time for customers would be reduced, as a portion of the repackaged produce would be served from the redistribution center rather than from the place of origin. In other words, a product that would otherwise be wasted could be sold again.
- It is important for suppliers to maintain a strategy of fast and flexible service [23]. Thus, suppliers could store produce in advance, close to the final demand and based on their estimates to serve customers throughout Europe as they receive orders. As a result, response times to customers’ orders (transit times) would be reduced by separating, albeit by a few days, transportation to the logistics center and the final shipment to the customer. Consequently, both service and customer loyalty would be improved by optimizing the cross-docking process. In the context of this strategy, supply time and its adaptation to demand would be a critical point to consider as we deal with products not apt for long storage periods [25]. This strategy would also have positive consequences on the reduction of waste both in storage centers at the place of origin and at retail points of sale (by increasing their shelf life).
- Existence of a key customer (modern distributor, e.g., Aldi or Lidl) with points of sale throughout Europe that would want to have a distribution center supplied by a priority place of origin (southeast Spain in our case). This strategy would be framed within an intensive supplier–customer collaboration in an ad hoc supply chain [26] that is easier to implement than the previous strategy (which assumes the existence of multiple customers and a more difficult demand to anticipate).
- At present, most shipping is organized by customers. This system would thus constitute a proactive strategy by suppliers, generating more stable relationships with customers.
- Improved capacity to attract more small retailers that require a more continuous service.

**Figure 2:**
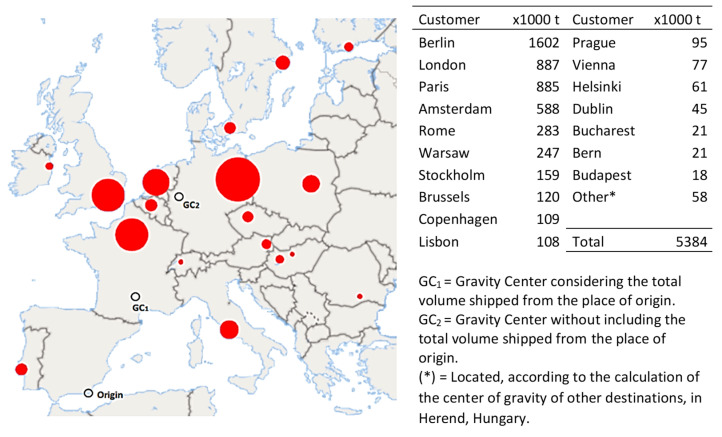
Main customers of fruit and vegetable exportation from southeast Spain. Source: own elaboration.

Figure 2 displays the location of the final exportation destinations of southeast Spain (total shipment of 5.4 million tons, valued at 5300 million €). The map in this figure also marks the center of gravity (GC) as the staging point calculated according to Euclidean distances [27]. When considering transportation from the place of origin of all the produce, GC_1_ would be located in France near Toulouse–Montpelier–Marseille, and if only the customers’ locations are considered, GC_2_ would be near Cologne. Currently, 98% of the shipping is carried out by means of refrigerated trucks using logistics-based multi-stop truckload shipping. This aspect is relevant because the customer (modern large distribution company) has included the European transport strategy [28] in its plans to promote the use of intermodality and reduce the environmental impact of transport [29]. For this reason, this article, after analyzing the optimal locations of redistribution centers, studied the possibility of supplying them through an intermodal option. With this aim, the multicriteria optimization [30,31] was introduced in the P-M and P-C methodology, utilizing transit time and shipping costs as decision variables.

## 2. Materials and Methods

The problem with p-medians (P-M) is locating p facilities in a network, minimizing the sum of all costs or distances from a demand point to the nearest facility, while respecting its full capacity. This problem has been widely addressed in the literature, namely, the seminal works of Hakimi [32]. The general formula implies:

(1) $$Min\;\underset{i}{\sum }\underset{j}{\sum }h_{i}d_{ij}Y_{ij}$$

Subject to:

(2) $$\underset{j}{\sum }Y_{ij}=1\;\forall i$$

(3) $$\underset{j}{\sum }X_{j}=p$$

(4) $$Y_{ij}-X_{j}\leq 0\;\forall i,j$$

(5) $$X_{j}=0,\;1\;\forall j$$

(6) $$Y_{ij}=0,\;1\;\forall i,j$$

where *Y_ij_* is 1 if customer *i* is served by facility *j*, 0—if not; *h_i_* is the demand at location *i*; *d_ij_* is the distance from location *i* to location *j*; *p* is the number of facilities to be located. The objective function (1) minimizes the demand-weighted distance between each demand node and the nearest facility. *X_j_* is 1 if a facility is located at candidate site *j*, 0—if not.

The first constraint (2) ensures that each demand node is allocated only to one facility. The second constraint (3) sets the number of facilities to open to exactly *p*. The next constraint (4) prevents demand from being allocated to candidate sites that do not have facilities. The last two constraints ensure that *X_i_* and *Y_ij_* have Boolean values of 0 or 1.

The objective function for the gravity p-median model (GP-M) is similar to P-M but the quantity of demand at node *h_i_* that is served by facility *j* is *A_j_h_i_*:

(7) $$Min\;\underset{i}{\sum }\underset{j}{\sum }A_{j}h_{i}d_{ij}Y_{ij}$$

Subject to constraints (2) to (6).

In the previous function, there is a new term, $A_{j}=\frac{\;u_{ij}}{\sum_{j}u_{ij}}$, specifying the probability that a customer residing at demand point *i* (demand located at node *i*) is served by facility *j* [33], where *u_ij_* is the utility of a facility located at node *j* for a customer originating at node *i*. The model assumes that $u_{ij}=\alpha_{j}d_{ij}^{-\lambda },$ where $\alpha_{j}$ denotes the attractiveness of facility *j* for customer *i* and *λ* denotes the parameter of the exponential distance decay function. In this case, *α_ij_* = 1 was utilized (common in the literature) as we dealt with a centralized scheme in which customers do not decide on the location. In other words, the utility measure decreases with distance and increases the attractiveness of a facility. The larger the value of lambda, the more attached the customer is to the nearest facility. In our case, we utilized *λ* = 0.6 to prioritize facilities near the customers as we dealt with highly perishable products. In practical terms, priority is given to the most centrally located points.

Finally, the p-center (P-C) problem consists of locating a specific number of facilities (p) while attempting to minimize the maximum distance at which a user of the nearest facility is located. The formula is as follows:

(8) Min W

Subject to:

(9) $$\underset{j}{\sum }Y_{ij}=1$$

(10) $$\underset{j}{\sum }X_{j}=p$$

(11) $$Y_{ij}-X_{j}\leq 0\;\forall i,j$$

(12) $$W\geq \underset{j}{\sum }h_{i}d_{ij}Y_{ij}\;\forall i$$

(13) $$X_{j}=0,\;1\;\forall j$$

(14) $$Y_{ij}=0,\;1\;\forall i,j$$

where an additional decision variable called W is included, which is the maximum distance between the demand location and the location of the intermediate facility that supplies it. The parameters and the decision variables of the model have the same definition as in the p-median model. As a novelty, restriction (12) forces W to be equal to the maximum distance.

It must be noted that the nodes (or possible intermediate facilities) are the capitals of the countries, which are customers of fruit and vegetable exportations supplied by southeast Spain (taken as the lone place of origin). The capital cities were chosen as the exact delivery points were unknown, although they would presumably be located near population agglomerations (capitals). The calculations were made using real distances in kilometers of the current road network obtained from Google Maps (a summary can be seen in Appendix A).

Regarding other aspects, the long-term instability intrinsic to the fruit and vegetable sector (e.g., increased trend of competition, climate change, etc.) leads us to consider an evolution of the p-median and p-center model redefining term *A_j_* introduced into the gravity p-median model in such a way that $A_{i}=\frac{h_{ij}^{p}}{h_{ij}}$, where $h_{i}^{p}$ is the forecast long-term demand for each of the customers. Thus, the objective function in the p-median is not $Min\;\sum_{i}\sum_{j}h_{i}A_{i}d_{ij}Y_{ij}$ = $Min\;\sum_{i}\sum_{j}h_{i}^{p\;}d_{ij}Y_{ij}$.

In the present case, in order to calculate $h_{i}^{p\;},$ we utilized the variation in demand growth for each destination in the previous five years. This methodology is reasonable considering that location decisions affect companies in the long term and are difficult to reverse.

The programming problems were solved using a substitution algorithm outlined in [34]. This heuristic is seeded in the initial list of p facilities. The algorithm attempts to replace each facility on the list with every possible candidate. If one of these replacements results in a better performance of the objective function, the replacement facility is substituted by a new one and the process begins anew. This continues until the algorithm determines the list of facilities that cannot be improved by replacing any facility on the list with any other candidate site.

### 2.1. Random Distances

In addition, in order to test robustness of the unloading points, we modified the models utilized and assumed that the distances were random variables (P-M^R^ and P-C^R^). This adjustment offered flexibility to the decision on whether to use country capitals as destination points. The new distance utilized was $d{\left(\xi \right)}_{ij}$, where $\xi_{ij}$ is the uncertain degree of the distance between i and j.

The resolution algorithms for this type of problems are complex and depend on the type of distributions involved, the treatment of random variables, whether the latter only appear in the objective function, only in the restrictions set, or in both [35]. In certain cases, the problem is notably simplified if the distribution of random variables assumes a specific probability distribution (normal is the most common), or if the initial objective function is linear, as in the present case. Our model seeks a deterministic equivalent of the stochastic problem [36,37], which can be resolved with the previously described method for the classic P-M and P-C problems.

The chosen approach represents an adaptation of the average variance model or value at risk [38,39]. The new formula in the case of the P-M model is:

(15) Min(∅)

Subject to:

(16) Prob [D(ξ)<∅]=α

where the decisionmaker sets probability α of fulfilling objective (∅) and the program determines the strategies that will help to achieve the objective with the given probability. $D\left(\xi \right)=\sum_{i}\sum_{j}h_{i}Y_{ij}d{\left(\xi \right)}_{ij}$, assuming all distances $d{\left(\xi \right)}_{ij}$ were normally distributed variables N($u_{ij}$,$\sigma_{ij}^{2}$), then $D\left(\xi \right)\sim N(\bar{D}\left(\xi \right)$, $\sigma_{D\left(\xi \right)}^{2}$). Additionally, the distances were considered independent random variables. Standardizing and reordering, $d{\left(\xi \right)}_{ij}=\sigma_{ij}\theta_{\alpha }+{µ}_{ij}$, $D{\left(\xi \right)}_{ij}=\sigma_{D\left(\xi \right)}\theta_{\alpha }+\bar{D}\left(\xi \right)$, with $\theta_{\alpha }\sim N(0$, 1), where $\theta_{0,9}$ = 1.30 (α=90%), it is possible to calculate probability in (13). Thus, a new deterministic objective function (P-MR) can be reformulated as:

(17) $$Min{\left[\sum_{i}\sum_{j}{\left(h_{i}\sigma_{ij}\right)}^{2}\right]}^{\frac{1}{2}}Y_{ij}\theta_{\alpha }+\sum_{i}\sum_{j}{µ}_{ij}h_{i}Y_{ij}$$

Formula (13) can be extrapolated to the P-C model and is easy to incorporate into solution algorithms. To solve the problem, we considered $u_{ij}$ = *d_ij_* and deviation (*σ*) to be equal to the existing distance between the capital and the second most populated city in the country: for example, in France, the distance between Paris and Marseilles; or between Berlin and Hamburg in Germany. Distances $d{\left(\xi \right)}_{ij}$ between the place of origin and the rest of the *j* destinations are displayed in Appendix A.

### 2.2. Multicriteria Optimization within the P-M and P-C Problems

The proposed models utilized the land distance travelled by trucks as a reference variable. However, it may be worthwhile to include other decision factors that introduce the possibility of a transport mode change in the modelling. The two most important variables that condition selection of a perishables transport system are time and cost [3], which is why they were introduced in a multicriteria optimization approach within the P-M and P-C models. In short, this paper sought to maximize the utility function as follows: $U=\sum_{p}^{n}w_{p}\;f_{p}\left(x\right),$ where $f_{s}\left(x\right)$ is the mathematical expression of the *p*th attribute and $w_{p}$ is the weight or pondering that the decisionmaker gives to that attribute. More specifically, the present study utilized weighted goal programming, which is a widely used multicriteria solution method in the literature [30,31]. This technique consists of minimizing deviations from the attributes in relation to an ideal point (unachievable), weighting the importance of each attribute for the decisionmaker. To calculate the weights, a matrix of payments was elaborated. The elements in the matrix of payments (Table 1) were obtained by optimizing each one of the objectives individually, such that f1n is the value of attribute “1” when objective “n” is optimized.

The following system of equations is solved below: $\sum_{p}^{n}w_{p}\;f_{p}\left(x\right)=f_{p}^{obs}$, where $\sum_{p}^{n}w_{p}=1$. Here, $f_{p}^{obs}$ is the observed value for the p^th^ objective (real value). Therefore, the problem that arises from a practical point of view is:

Max $\sum_{p}^{n}\frac{w_{p}}{K_{p}}f_{p}\left(x\right)$ subject to F∈ *x*; *x* ≥ 0,

where *F* is the set of the restrictions utilized and $K_{i}$ is the factor of standardization: the difference between the ideal value: the difference between the ideal value $f_{1}^{*}$ and the anti-ideal value $f_{1*}.$ Following the programming model’s application, one noteworthy characteristic that arose was that there was no observed value $f_{p}^{obs}$ available for the actual situation. This was because the land transport system is practically the only one currently used and the intermodal routes available are not in service. Therefore, a “future” observed value was utilized, $f_{*}^{obs}$, which implied that a decisionmaker was willing to lose time on the delivery in favor of the savings on costs. Based on this premise, sensitivity analysis was conducted varying the value of $f_{*}^{obs}$.

In order to create the P-M or P-C multicriteria model, two objective functions must be utilized: one for the cost and another for the transport time:

$f\left(c\right)=\sum_{i}\sum_{j}h_{i}c_{ij}d_{ij}Y_{ij}$, where *c_ij_* is the transport cost (€/kg) from location i to location j;

$f\left(t\right)=\sum_{i}\sum_{j}h_{i}t_{ij}Y_{ij}$, where *t_ij_* is the time (hours) to transport goods from location i to location j.

For this purpose, Equation (1) was modified in the case of P-M, Equation (12)—in the case of P-C.

## 3. Results and Discussion

**Figure 3:**
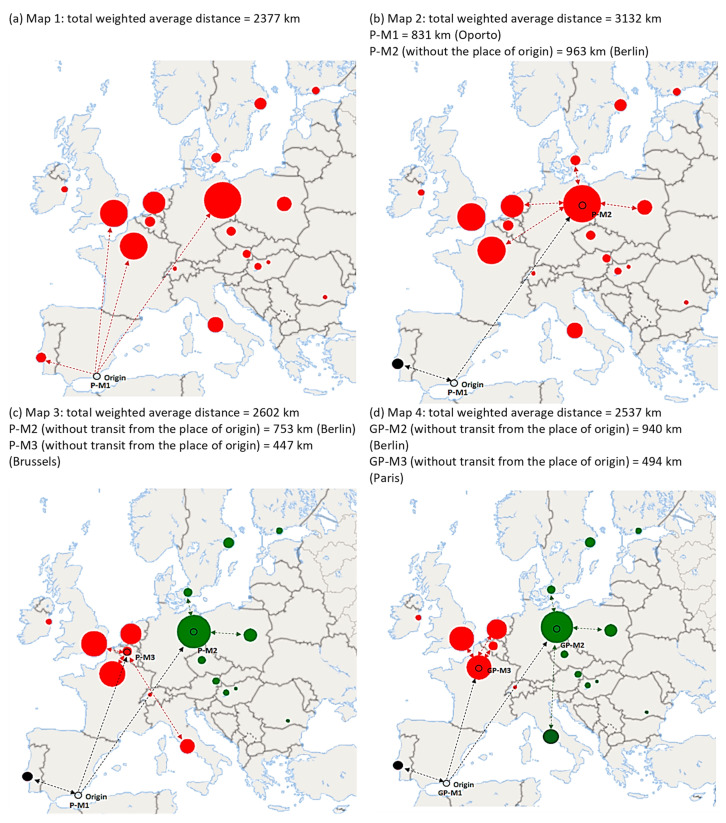
Results of the p-median (maps **a**–**c**) and gravity p-median models (map **d**). Lines: only samples of routes are shown. Each color represents customer allocation by the redistribution center. Source: own elaboration.

Figure 3 displays the results of the p-median model (P-M) and the gravity p-median (GP-M). All the models included the shipment of merchandise from the place of origin to the intermediate facilities calculated. Considering *p* = 1 (Figure 3a), that is, a single redistribution center, the model showed the current location. Initially, it is preferable that the logistics center be located at the place of origin. The average weighted distance of each shipment was nearly 2400 km, approximately 32 h of transit time per truck. It is worth highlighting that 98% of all shipments are currently made by means of refrigerated trucks.

**Figure 4:**
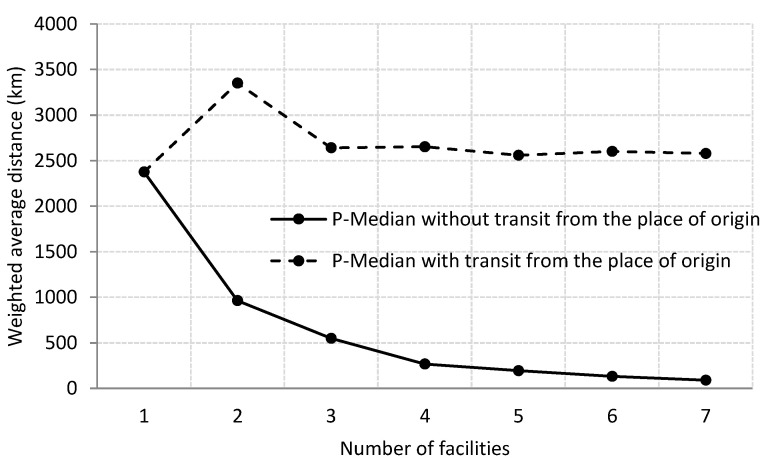
Median tradeoff curve. Source: own elaboration.

When considering *p* = 2 (Figure 3b), the place of origin remained the distributor for Portugal (Lisbon), while Germany (Berlin) became the location of the second center. It must be noted that the total weighted distance significantly increased (41% in relation to the initial location). However, if improving the customer service is considered a priority objective, the distance traveled to fulfill an order would only be 963 km if supplied from Berlin (Germany). If *p* = 3 (Figure 3c), an additional optimum location in Belgium (Brussels) would be added to the place of origin and Berlin to supply the United Kingdom, Ireland, the Netherlands, France, Italy, and Switzerland. Using center 1 at the place of origin and 2 at the destination is better than the initial situation in terms of delivery routes considering shipments from the place of origin (9% more). However, merchandise shipments (and times) to fulfill customer orders would be significantly reduced: falling to 447 km on average if supplied from Brussels and to 753 km if shipped from Berlin. Undoubtedly, this strategy (a center at the place of origin and two at destinations) is better as it optimizes the objectives of the search for a consolidation facility. In fact, by observing the median tradeoff curve (Figure 4), a new center would neither reduce the total distance travelled nor the weighted response distance to the customer.

The last of the maps above (Figure 3d) is the result of applying the gravity p-median (G-PM) methodology considering *p* = 3. This method, as previously mentioned, may favor locations with the presence of an agglomeration of destinations near the customer (λ = 0.6), which is convenient for perishable products. The key change is that the second redistribution center would be in Paris (France). This system prioritizes redistribution centers located in areas with the highest demand. The total shipping distance improved with respect to the P-M model (2537 vs. 2602 km); however, the distances of quick response to customers would not be shorter. This method appears to polarize the strategies. Thus, it could prove to be a good decision-making system, taking into account the changeability of the sector, that is, the variability of short- and medium-term demand as the G-PM method considers different factors when selecting a location. This characteristic appears to coincide a priori with the p-center methodology.

**Figure 5:**
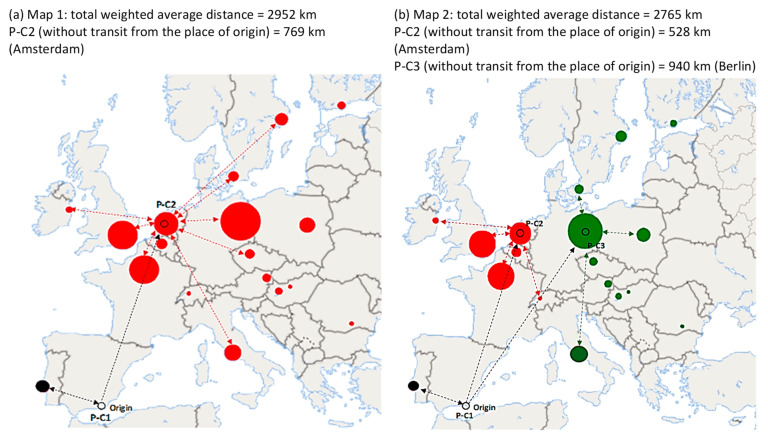
Results of the p-center model (*p* = 2 and *p* = 3). Lines: only samples of routes are shown. Each color represents the customer allocation by the redistribution center. Source: own elaboration.

Considering model *p* = 2, the redistribution center (Figure 5a) should be located in the Netherlands (Amsterdam), albeit the place of origin still supplies Portugal. This method minimizes the maximum distance of the distribution center, favoring more balanced locations. What this means is that if we wish to make average transport distances shorter (necessary with perishables), a location in this area could be suitable. This strategy could prove ideal for a key customer interested in having its own redistribution center. The total weighted distance (2952 km) is shorter than that of the existing *p* = 2 median model (3132 km). Furthermore, the response time to customers is better (769 km vs. 963 km). This option is good when insufficient funds are available to have a third distribution center. For the *p* = 3 center (Figure 5b), a center in Berlin would be added to that of Amsterdam, but this would not make any positive contributions in comparison to the other options.

**Figure 6:**
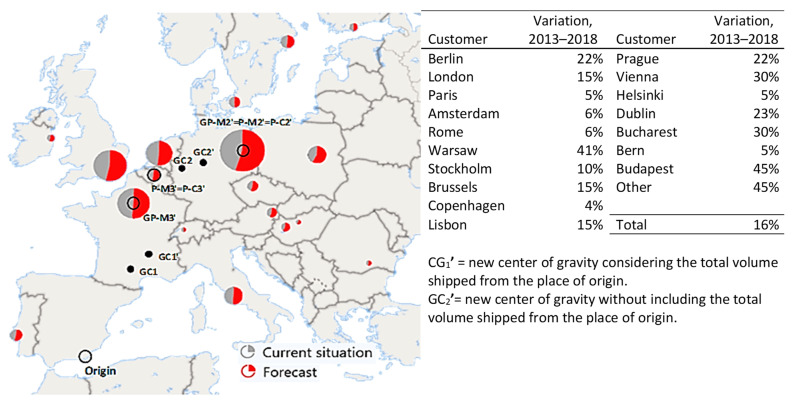
Summary of results applying variations in demand to the gravity p-median (GP-M’), p-median (P-M’), and p-center models (P-C’) for *p* = 3. Source: own elaboration using the [1] data.

The recalculation of the models P-M’ and GP-M’ utilizing the new forecast demands provided similar results to the previous data (Figure 6): the *p* = 2 median and *p* = 3 median models (and the gravity p-median) would be equal to the previous ones without applying variations (Figure 3, Maps 3 and 4). The strength of the model against long-term demand changes was corroborated. The new p-center (P-C’) model had some changes. The new *p* = 2 centers would be located at the place origin and in Berlin rather than in Brussels. The new *p* = 3 centers would be located at the place of origin, in Brussels (rather than Amsterdam), and in Berlin. The centers of gravity would move to the north and east of Europe, coinciding with the increase in demand from countries such as Germany and Poland and the stabilization of French purchases.

**Figure 7:**
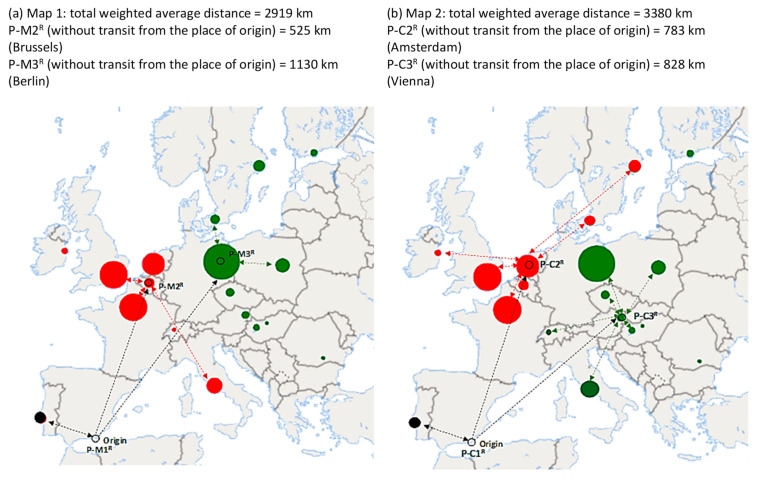
Results of the p-median (map **a**) and p-center models (map **b**) with random distances (*p* = 3). Lines: only samples of routes are shown. Each color represents the customer allocation by redistribution center. Source: own elaboration.

When the random P-M^R^ model was utilized considering *p* = 3, the optimal locations were the original ones (Figure 7a): place of origin, Brussels, and Berlin. Furthermore, the robustness of the results is also revealed by considering random deviations at the unloading points. However, the total weighted distance would increase due to the use of new distances that include possible deviations (specifically, 11%). The random P-C^R^ model for *p* = 3 (Figure 7b) underwent modifications with respect to the original. When including possible deviations, the total weighted distance would increase by 21% compared to the result calculated with deterministic distances. In general, the random models incorporate, with respect to the original ones, the uncertainty at unloading points (even including changes once the shipment process has started), an aspect that may greatly influence decision-making when choosing redistribution centers.

### Intermodal Option

Another aspect worth studying is whether this location strategy would be compatible with intermodal transport including rail freight or even Short Sea Shipping (SSS). In addition to this point, we sought to identify what the balance between cost and time would be (loss of quality and increased waste) for the exporter in order to favor a change to the transportation modality.

Regarding the SSS use, although usually cheaper than by road, transit times are longer [40,41]. This point is crucial when transporting perishable products where product quality can be adversely affected. In any case, this variable is also influenced by frequency and freight volume. It is possible that as the willingness to use intermodal shipping increases, the frequency of boats per week will increase as well, thereby reducing the time difference between intermodal and land transport and the unit cost. Several studies have analyzed the possibility of creating ad hoc lines between the Spanish southeast and Northern Europe for the transit of perishables [24]: the optimal ports on the Atlantic side (Rotterdam and Dunkirk) are consistent with the existence of redistribution centers (GP-M, P-M, and P-C) for the whole Europe or only one for supplying western Europe (France, the United Kingdom, Belgium, and the Netherlands, including distribution in the Lisbon route).

Approached from a medium- and long-term perspective, the option of using trains as an alternative means could be viable with the full completion of the so-called “Mediterranean Corridor”—a railway line that would link Algeciras (southeast Spain) with Perpignan (southeast France), running along the entire Spanish coast. Currently, there is no project under way to unite this Corridor; there is simply a series of lines with double rails, third tracks or independent tracks with completely different projects and deadlines. In any case, the current option is to connect the finished sections of the Mediterranean, Central, and Northern Mediterranean Corridor, which includes Rotterdam. Furthermore, the railway option continues to prove deficient in terms of transportation of perishable goods [42]; it requires large-scale cargo groupage, which slows down journeys [43], and it lacks suitable cold storage infrastructure. In summary, the proposed distribution centers could be loaded by road, SSS, and rail freight in accordance with the European transport strategy to promote transport mode balance and reduce CO_2_ emissions, especially in the case of perishables [44].

As an example, Table 2 shows the results for the intermodal options using the P-C2 location (the closest to the Rotterdam port): road use is the only viable option due to its flexibility and transit time (eight times faster than the other options). Thus, in practice, this last point nullifies the advantages of cost and CO_2_ reduction. In any case, the use of specific lines for the transport of perishables could reduce the transit times of SSS and rail freight by more than 50%, although today this option, in the case of trains, is unrealistic [45].

Taking the two most viable options from Table 2, (i) intermodal transport via Rotterdam vs. (ii) land transport, using Brussels in both cases as the redistribution center, the P-C2 multicriteria model was calculated, utilizing time and transport costs as decision factors. From the data presented in Table 2, it is possible to extract the values with which to calculate the matrix of payments (Table 3).

The sensitivity analysis (Table 4) revealed the difficulty that comes with changing the mode of transport. For example, below is a scenario for taking the current situation in which the use of intermodal transport is zero and we want it to become acceptable for the marketing company. In this case, the sector would have to be willing to accept a 114% increase in the merchandise shipping time, but would benefit from the advantage of a 29% cost reduction. This means that the decisionmaker would have to value the cost factor 6.7 times more than the time factor. The current situation arises from the fact that the operator believes maritime transport involves greater risk due to less control of merchandise on board and a greater number of losses (wastes) due to prolonged transit of highly perishable products. In other words, the risk that products may fail to arrive in perfect condition to customers and assuming that they must be discarded impedes the change to more sustainable transport systems. If we assume that the losses due to poor quality are proportional to transport time, these losses, at best, would account for 30% of all the shipments, which is a completely unsustainable scenario.

## 4. Conclusions

This work attempted to contribute to the debate on the practical utilization of different location models for consolidation, redistribution, or repackaging centers in a supply chain network in order to reduce waste (costs), improve service quality, and increase the shelf life of products for the final consumer. Within this framework, multicriteria techniques were also applied to ascertain the optimum weight between transport cost and transit time which favors the balance between land and intermodal options. For this purpose, an analysis was carried out for the specific case of shipping perishables from southeast Spain (the main European supplier) to its key customers. This case is interesting in that it requires taking into consideration differential aspects with regard to non-perishable merchandise, for example, the need for agile response time to customers. This strategy is in accordance with that implemented by the main customers, the large European retailers. They use different strategies depending on the type of food [49] and try to collaborate by making logistics centers with suppliers in order to implement new consumer distribution strategies [25,28,50]. In addition, it is possible to ensure additional supply lines in case of any contingencies. On the other hand, from the point of view of administration, the EU recognizes that food waste is a problem along the entire food supply chain and, therefore, action should be targeted at all stages. Such measures could result in potential benefits for all those involved by emphasizing prevention and exploiting economic opportunities [51]. In fact, in the case of agricultural products, the common agricultural policy (CAP) addresses the subject of food waste by incorporating the circular economy point of view of the ‘Farm to Fork’ EU strategy, specifically in the purposes of more sustainable processing and farm transport, healthy consumption, and FLW reduction. In more general terms, waste management in the perishable supply chain is a great opportunity to help achieve the Sustainable Development Goals (SDGs) promoting efficient distribution and reuse from farm to fork and post-consumer in a framework of collaboration and partnership.

In theoretical terms, it is demonstrated that the gravity p-median model can generate small advantages when the nature of merchandise requires closer proximity to customers, at centrally located points, and in contexts of uncertain short-term demand, as is the case of the fruits and vegetables analyzed. In general, it is demonstrated that each model (p-median, gravity p-median, and p-center) has distinct characteristics which make it ideal depending on the peculiarities of the product and operating strategy. In addition, the strength of the p-median was tested in relation to the long-term changes in demand conditions, corroborating greater robustness in the case of the p-median model, even considering randomness in distances. However, the p-center model is highly variable to changes in the normal conditions of quantities and distances. The formulation of models introducing long-term demand forecasts and the randomness in some variables can prove useful as location decisions condition companies in the long term and are difficult to reverse.

Regarding the solution to the empirical problem of creating a fruit and vegetable distribution center in the middle of Europe to improve the waste reduction strategy in shipments, various options can be proposed. If the decision were made to create a single center at the destination, the ideal location would be the Netherlands. This scenario is validated considering the present reality as this country displays a proven capacity for re-exportation. If two centers were to be established at the destination, Belgium would supply the west and south of Europe and Germany would distribute to the east and north of Europe. As for using the intermodal option for supplying these facilities, this would involve great difficulty, mainly due to the transit time (critical point for perishables), which in practice nullifies the cost advantages and reduced environmental impact. However, this last point may carry a certain degree of contradiction: although intermodality would reduce CO_2_ emissions, it would increase the chances of having a disposable product at destination. In any case, the creation of redistribution centers could favor the transport mode change, provided that ad hoc lines (much faster) are created. In addition, the use of intermodality will depend on the implementation of initiatives that foster a change in the perception of exporters (e.g., the poor image of SSS for door-to-door service) and favor willingness to utilize ships as a transport system (e.g., eradicate the lack of knowledge of this system among many exporters). At the same time, it would be necessary to improve the systems for preserving merchandise in order to avoid waste on ships and at cross-docking points.

It is worth highlighting that this work is the first to explore this subject as applied to a real case, and such research is necessary to maintain competitiveness of this key export sector in Spain. This study has not contemplated the cost of the proposed facilities, the need to establish a packing plant near the customer, or even part of production. These aspects deserve their own exclusive analysis. Please note that the proposed distribution strategy would require “refinement” of cross-docking procedures, e.g., the use of different transportation processes, temporary inventories, or combining with other merchandise [49]. Ultimately, these strategies require a collaborative effort to be made by commercialization companies at the place of origin, an aspect which makes their implementation more complicated.

## Figures and Tables

**Table 1:** Matrix of payments.

	*f*_1_	*f*_2_	…	*f_n_*
*f*_1_	$f_{1}^{*}$ = *f*_11_	*f*_12_	,,,	*f*_1*n*_
…	…	…	…	…
*f*_*n*_	*f*_*n*1_	*f*_*n*2_	…	*f*_*nn*_

**Table 2:** Examples of the results for intermodal transport for P-C2 (Figure 5, map 1).

**SSS–Road**	**SSS, Southest Spain–Rotterdam**	**Road, Rotterdam–Brusells**	**Road, Brusells** **–End Clients**	**Total**	**% Variat. on the Road**
Km	2740	143	545	3428	24
Time (h) (1)	240	1.4	5.0	246	782
Time (h) (2)	106	1.4	5.0	112	304
Cost (€/kg) (1)	0.10	0.01	0.02	0.13	–38
Cost (€/kg) (2)	0.19	0.01	0.02	0.22	6
tCO^2^ (trip)	1.10	0.23	0.87	2.20	–50
**Train–road**	**SSS, southest Spain–Rotterdam**	**Train, southest Spain–Brusells**	**Road, Brusells–end clients**	**Total**	**% variat. on the road**
Km	-	1610	545	2155	–22
Time (h) (1)	-	240	5	245	775
Cost (€) (1)	-	0.16	0.02	0.18	–14
tCO^2^ (trip)	-	1.61	0.87	2.48	–44
**Road**	**SSS, southest Spain–Rotterdam**	**Road, southest Spain–Brusells**	**Road, Brusells–end clients**	**Total**	
Km	-	2210	545	2755	-
Time (h)	-	23	5.0	28	-
Cost (€) (3)	-	0.19	0.02	0.21	-
tCO^2^ (trip)	-	3.53	0.87	4.41	-

(1) = regular line (door to port); (2) = line made ad hoc (from port to port from [22]); (3) = round trip. Source: own elaboration from [46,47,48].

**Table 3:** Values to create the matrix of payments between cost and time.

	Cost (€/kg)	Time (h)
Cost (€/kg)	0.13 *	72
Time (h)	0.21	28 *

(*) Values when that same variable is optimized (ideal value).

**Table 4:** Sensitivity analysis.

Values Tested in the Sensitivity Analysis		Importance the Decisionmaker Gives to the Variables Based on the Tested Values:		Real Results		Calculated Results
Cost (€/kg)	Time (h)	Weight (Cost)	Weight (Time)	Cost (€/kg)	Time (h)	With the p-Center Model (Two Facilities)
0.13	72	100%	0%	0.13	72	Brusells (I)
0.15	60	87%	13%	0.13	72	Brusells (I)
0.16	52	81%	19%	0.21	28	Brusells (T)
0.17	45	52%	48%	0.21	28	Brusells (T)
0.19	35	23%	77%	0.21	28	Brusells (T)
0.21	28	0%	100%	0.21	28	Brusells (T)

(I) = intermodal via Amsterdam; T = using trucks.

## Data Availability

Not applicable.

## Appendix A

**Table A1:** Distance summary in kilometers.

Destination	d = from Southest Spain to:	Average Weighted (*)	d(ξ) = from Southest Spain to:
Berlin	2403	726	2780
Paris	1820	840	2828
London	2221	900	2481
Amsterdam	2210	729	2353
Warsaw	3120	1191	3608
Rome	2504	1580	3309
Stockholm	3404	1567	3814
Prague	2612	882	2879
Lisbon	831	2481	636
Copenhagen	2902	977	3145
Brussels	2012	717	2090
Vienna	2803	1095	2907
Helsinki	4298	2162	4532
Bucharest	3499	2050	4210
Bern	1982	988	2021
Dublin	2601	1474	2930
Budapest	3006	1310	3305
Herend	2268	1104	2639
Origin:Southest Spain		2377	

(*) With respect to the rest of the destinations and weighted according to demand. Source: own elaboration.
